# Improved outcomes in metastatic germ cell cancer: results from a large cohort study

**DOI:** 10.1007/s00432-020-03343-2

**Published:** 2020-08-09

**Authors:** Marcus Hentrich, Jessica Debole, Vindi Jurinovic, Arthur Gerl

**Affiliations:** 1 Department of Hematology and Oncology, Red Cross Hospital Munich, Ludwig-Maximilians-University Munich, Nymphenburger Str. 163, 80634 Munich, Germany; 2 Private Practice of Oncology, Munich, Germany; 3 Helios Klinikum München West, Munich, Germany; 4 Institute of Biometrics and Epidemiology, Ludwig-Maximilians-University Munich, Munich, Germany

**Keywords:** Germ cell cancer, Germ cell tumors, Seminoma, Non-seminoma, IGCCCG classification

## Abstract

**Purpose:**

Treatment of metastatic germ cell cancer (GCC) is based on the International Germ Cell Cancer Collaborative Group (IGCCCG) prognostic classification published in 1997. 5-year survival rates were reported to be 91%, 79%, and 48% for patients with good, intermediate and poor prognosis, respectively. However, treatment results may have improved over time due to cumulative experience, improved supportive care and modern-type chemotherapy.

**Methods:**

Patients with metastatic GCC who received cisplatin-based chemotherapy at two institutions in Munich between 2000 and 2013 were retrospectively studied. Clinical characteristics, treatment and outcomes were analyzed with respect to the IGCCG prognostic classification.

**Results:**

Of 225 patients (median age 35 years), 72 (32%) had seminoma (S) and 153 (68%) nonseminoma. 175 (78%), 30 (13%) and 20 patients (9%) had good, intermediate and poor prognosis according to the IGCCCG classification. The 2-year-progression free survival of patients with good, intermediate and poor prognosis was 91%, 83% and 37%, and the 5-year-overall survival (OS) was 98%, 96%, and 66%, respectively. There was no significant difference in the OS between patients in the good and intermediate prognosis group.

**Conclusion:**

Compared to data from the original IGCCCG classification system, the outcome of patients with metastatic GCC has considerably improved over time. While the prognosis of intermediate-risk patients is excellent, treatment in the poor-prognosis group remains to be improved.

## Introduction

Testicular germ cell cancer is the most common cancer in men aged 18–40 years with an estimated 4120 new cases detected in Germany in 2016 (Robert-Koch-Institute 2019). Today, a cure is expected in 95% of all patients diagnosed with testicular cancer and in approximately 80% of patients with metastatic disease (Hanna and Einhorn 2014). Treatment of metastatic germ cell cancer (GCC) is based on the 1997 International Germ Cell Cancer Collaborative Group (IGCCCG) prognostic classification (IGCCCG 1997; Beyer et al. 2013; Albers et al. 2015; Honecker et al. 2018). Three cycles of cisplatin, etoposide, bleomycin (BEP) combination chemotherapy have become standard of care for good-risk patients while four cycles of BEP are the reference for intermediate- and poor-risk patients (Beyer et al. 2013; Honecker et al. 2018). According to the IGCCCG cohort, five-year survival rates are 91%, 79%, and 48% for patients with good, intermediate and poor prognosis, respectively (IGCCCG 1997). However, the IGCCCG classification is based on treatments applied between 1975 and 1990. Recent data indicate that the outcome of patients with metastatic GCC may have improved over time due to cumulative experience of treating physicians, improved supportive care and modern-type chemotherapy (van Dijk et al. 2006; Raggi et al. 2015). The aim of the present study was to analyze the outcome of patients with metastatic GCC treated between 2000 and 2013 at two experienced institutions in Munich, Germany. We presumed that the outcome of metastatic GCC has considerably improved compared to the original IGCCCG experience.

## Methods

All patients who underwent first line chemotherapy between January 2000 and December 2013 at Private Practice of Oncology and at the Department of Hematology and Oncology, Harlaching Hospital, both located in Munich, were identified through the institutional database. Inclusion criteria were (1) pathology proven metastatic GCC, (2) age ≥ 17 years, (3) start of chemotherapy for metastatic GCC between January 2000 and December 2013, and (4) at least 2-years follow-up. Patients were excluded if data on tumor stage, treatment or follow-up were incomplete.

The medical records were reviewed with regard to age at time of initial diagnosis, histopathology, stage, tumor marker levels, metastatic spread, type and duration of chemotherapy, and outcome.

The primary endpoint was the 5-years overall survival (OS) rate. Secondary endpoints included progression-free survival (PFS) as well as PFS and OS of relapsed patients. The study was approved by the Ethics committee of Ludwig-Maximilians-University Munich. The study was performed in accordance with the Declaration of Helsinki.

### Statistics

PFS was measured from the beginning of the chemotherapy to the time of progression, relapse or death. OS was calculated from the beginning of the chemotherapy to last follow-up or to death from any cause. Patients without an event were censored at the date of last follow-up. The probability of PFS and OS was determined by the Kaplan–Meier Method and differences between subgroups of patients were assessed by the log-rank test. Statistical analyses including descriptive statistics such as frequency, mean, median, range, inter-quartile range, minimum and maximum were performed by using SPSS software (IBM SPSS Statistic, version 21.0). All p-values were two-sided. *P* values of 0.05 or less were considered statistically significant.

## Results

### Patient characteristics

Of 255 patients identified, 30 were excluded due to incomplete data. Main characteristics of the 225 patients included into the study are outlined in Table 1. The median age of the cohort was 35 years (range 17–66). Seminoma and nonseminoma were diagnosed in 72 (32%) and 153 (68%) patients, and 204 patients (91%) had a primary gonadal GCC. The cohort includes 56 patients managed with active surveillance for stage I disease who developed metastases after a median time of 9.5 months (range 0.5–15). According to the IGCCCG classification system, 175 (78%), 30 (13%) and 20 patients (9%) had good, intermediate and poor prognosis GCC as compared to 60%, 26% and 14% in the original IGCCCG cohort.

**Table 1:** Patients characteristics and first line treatment

Patients (*n*)	225
Median age	35 (17–66)
Pathology	
Seminoma	72 (32%)
Non-seminoma	153 (68%)
Primary location	
Gonadal	204 (91%)
Retroperitoneal	18 (8%)
Seminoma	9
Non-seminoma	9
Mediastinal	3
Non-seminoma	3
Metastatic sites	
Abdominal	180 (80%)
Lung	49 (22%)
Liver	9 (4%)
Bone	2 (1%)
Brain	4 (2%)
Other^a^	4 (2%)
Relapse from Stage I	56 (25%)
Median time to progression	9.5 month (range 0.5–15)
IGCCCG risk at relapse from Stage I	
Good	52
Intermediate	4
IGCCCG prognostic group (entire cohort)	
Good	175 (78%)
Intermediate	30 (13%)
Poor	20 (9%)

^a^Spleen, Intestine, Kidney

Type of first line chemotherapy and the number of cycles given are shown in Table 2. The vast majority of patients received three to four cycles of platinum-based chemotherapy, while primary high-dose chemotherapy (HDCT) was applied to three patients in the poor prognosis group. All nonseminoma patients with residual tumor > 1 cm after chemotherapy were routinely scheduled for secondary surgery. Thus, 33 of 225 (15%) patients underwent secondary retroperitoneal lymph node dissection (*n* = 28), and/or secondary surgery of the lungs (*n* = 5), liver (*n* = 1), mediastinal (*n* = 3) and infraclavicular lymph nodes (*n* = 1). Histopathological examination revealed necrosis/fibrosis in 16 of 38 (42%) resection specimens, teratoma in 10 (26%), vital GCC in 7 (18%), and malignancies other than GCC in 5 (13%) (spindle cell tumor [*n* = 3], undifferentiated non-small cell carcinoma [*n* = 1], not specified [*n* = 1]).

**Table 2:** First line treatment for metastatic GCC

Good prognosis (*n* = 175)	
3 × PEB^a^	159 (91%)
4 × PEB	9 (5%)
4 × PE	3 (2%)
Other^b^	4 (2%)
Intermediate prognosis (*n* = 30)	
4 × PEB	25 (83%)
2 × PEB— > 2 × VIP	1 (3%)
3 × PEB	4 (13%)
Poor prognosis (*n* = 20)	
4 × PEB	13 (65%)
1 × VIP—> 3 × PEB	4 (20%)
VIP + HDCT	3 (15%)

^a^Including PEB—> VIP (*n* = 3)

^b^PE × 3 (*n* = 3), PEB × 2 (*n* = 1)

Salvage treatment in relapsed patients was applied to 14, 5 and 11 patients of the good, intermediate and poor prognosis group, respectively. It consisted of surgery alone for teratoma in seven of 30 (23%) patients, all but one of whom are alive in ongoing remission. Conventional dose first salvage chemotherapy (CDCT) and HDCT were applied to 16 and 7 patients, respectively. CDCT regimens used were cisplatin, etoposide, ifosfamide (VIP, *n* = 13), cisplatin, ifosfamide, paclitaxel (TIP, *n* = 1), and gemcitabine, oxaliplatin, paclitaxel (GOP, *n* = 2). Second and higher relapses occurred in twelve of 30 patients (40%). Second salvage chemotherapy consisted of GOP (*n* = 2), gemcitabine, paclitaxel (*n* = 2), cisplatin, epirubicine (*n* = 1), oxaliplatin, paclitaxel (*n* = 1), oxaliplatin, irinotecan (*n* = 1), and HDCT (*n* = 5). Of the 12 patients undergoing second salvage chemotherapy, 6 died of GCC while the others are alive and well. Palliative treatment with everolimus was given to two patients within a clinical trial (Fenner et al. 2019).

### Survival

**Fig. 1:**
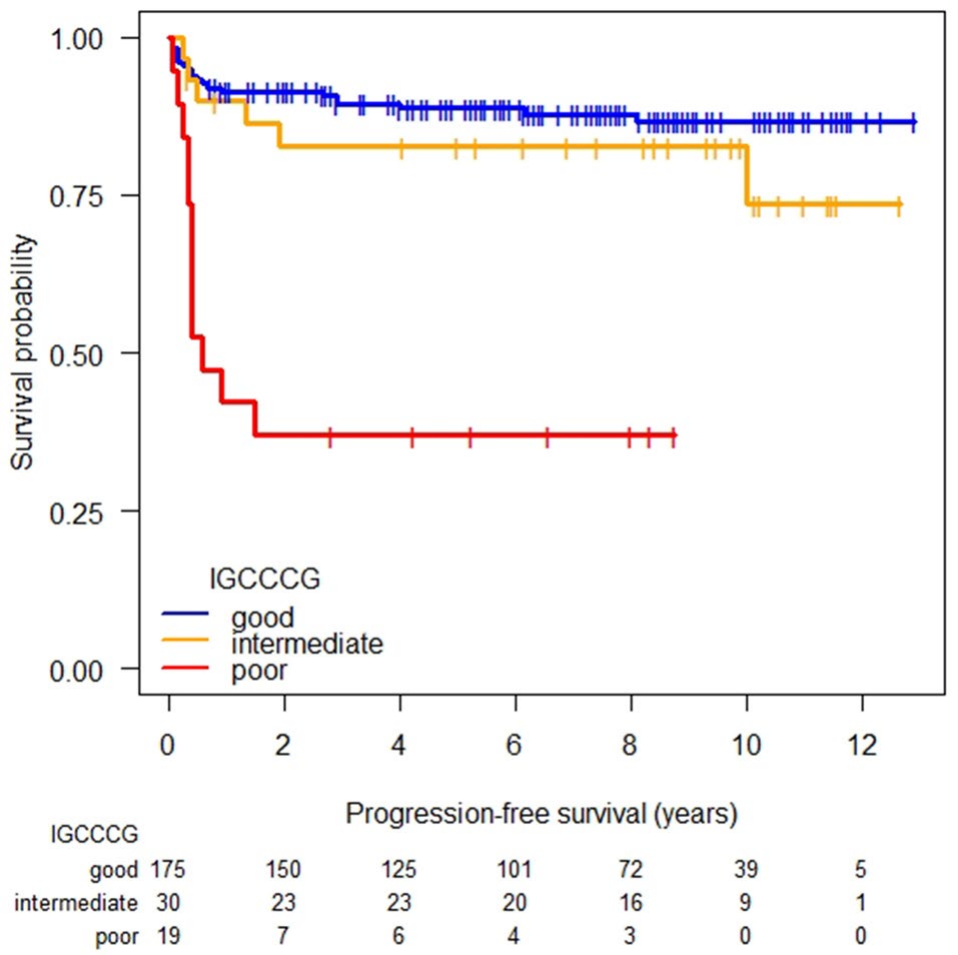
Progression-free survival according to IGCCCG prognostic groups

After a median follow-up of 95 months (95% confidence interval [CI], 88–104), the 2-year-PFS of patients with good, intermediate and poor prognosis was 91% (95% CI 87–96), 83% (95% CI 70–98) and 37% (95% CI 20–66) (Fig. 1). The corresponding 5-year-PFS of the original IGCCCG cohort has been reported to be 88%, 78% and 41%. In our analysis the difference between the good and poor prognosis group (*P* < 0.0001) and between the intermediate and poor prognosis group (*P* = 0.00045) was statistically significant. By contrast, the difference between the good and intermediate group was not statistically significant (*P* = 0.281).

**Fig. 2:**
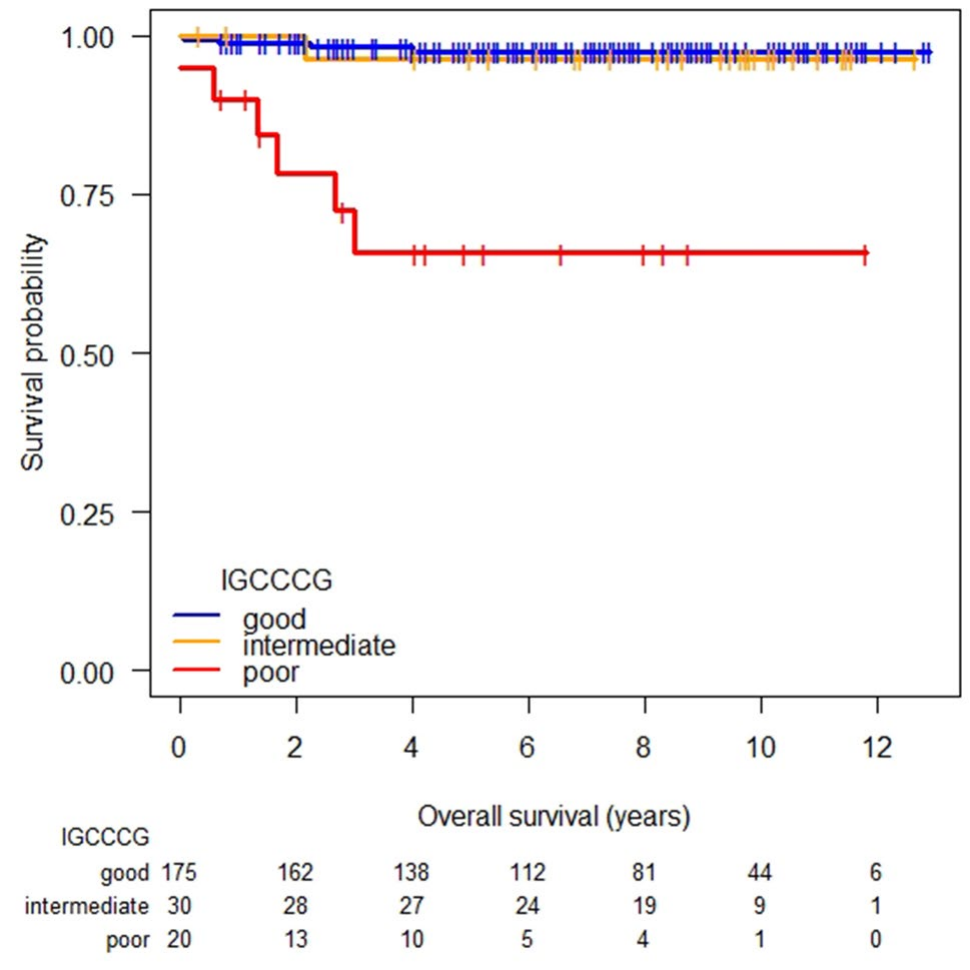
Overall survival according to IGCCCG prognostic groups

Overall, eleven of 225 (5%) patients have died, ten of which from GCC and one from community-acquired pneumonia unrelated to cancer treatment (Table 3). The 5-year-OS of our cohort was 98% (95% CI 95–100), 96% (95% CI 90–100), and 66% (95% CI 47–93), for the good, intermediate and poor prognosis group, respectively (Fig. 2; Table 3). This compares favorably to the 5-year-OS of 91%, 79% and 48% predicted by the original IGCCCG score. The corresponding mean survival times of our cohort were 152 (95% CI 148–155), 147 (95% CI 139–156), and 100 months (95% CI 72–128). There was no significant difference in the OS between patients in the good and intermediate prognosis group (*P* = 0.754). By contrast, the OS of patients from the good and the intermediate prognosis group was significantly superior to that of the poor prognosis group (*P* < 0.0001 and *P* = 0.004, respectively). Further, the 5-year-OS was significantly better in poor-risk patients without non-pulmonary visceral metastasis (NPVM) compared to patients with NPVM (5-year-OS 100% vs. 38%, *P* = 0.033).

**Table 3:** Outcome according to the IGCCCG risk classification

2-years PFS	
Good	91% (95% CI 87–96)
Intermediate	83% (95% CI 70–98)
Poor	37% (95% CI 20–66)
5-years OS	
Good	98% (95% CI 95–100)
Intermediate	96% (95% CI 90–100)
Poor	66% (95% CI 47–93)
Relapse/progression	30/225 (13%)
Good	14/175 (8%)
Intermediate	5/30 (17%)
Poor	11/20 (55%)
Death	11/225 (5%)
Good (*n*)	4/175 (2%)
Intermediate (*n*)	1/30 (3%)
Poor (*n*)	6/20 (30%)

**Fig. 3:**
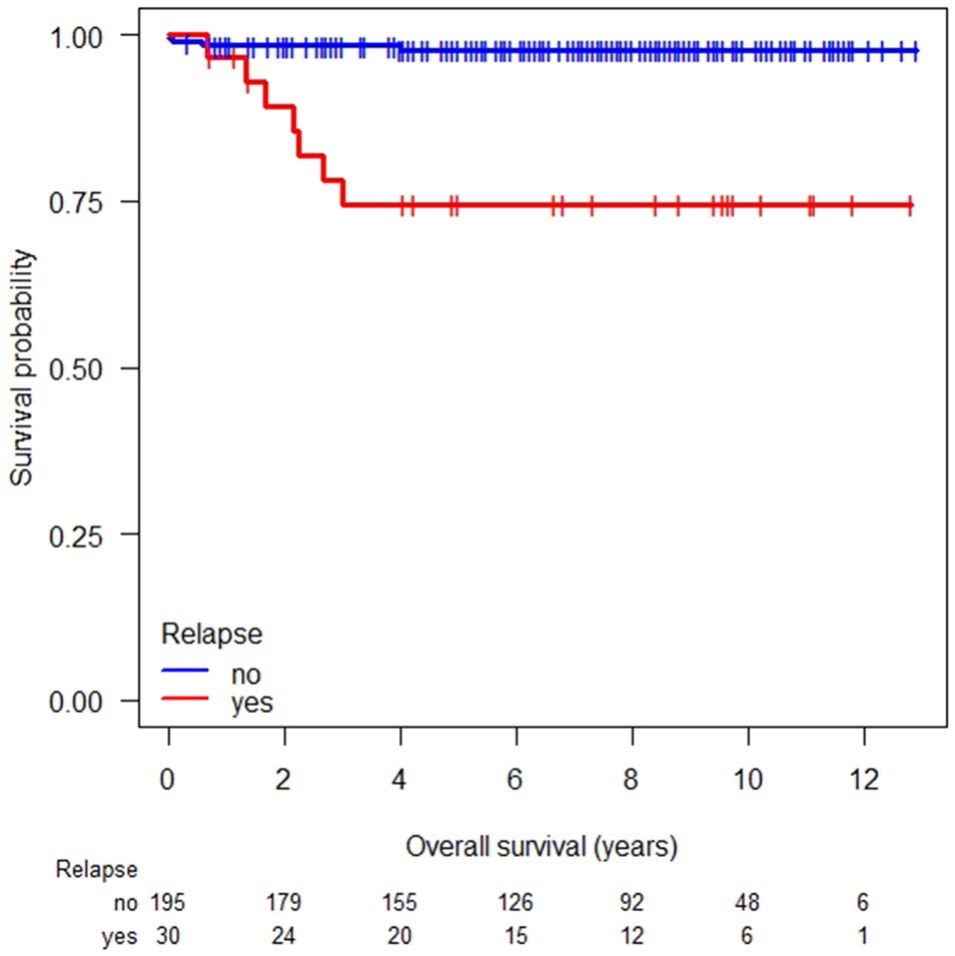
Overall survival in relapsed vs. non-relapsed patients

The cumulative 5-year-OS of relapsed patients was 75% (95% CI 60–93) without significant differences between the good, intermediate and poor prognosis group (Fig. 3) (*P* = 0.238). Of 21 patients treated with first salvage cisplatin-based CDCT (*n* = 14) or carboplatin-based HDCT (*n* = 7) the International Prognostic Factors Study Group (IPFSG) scores were very low (*n* = 2), low (*n* = 3), intermediate (*n* = 5), high (*n* = 6), and very high (*n* = 5), respectively (International Prognostic Factors Study Group et al. 2010). However, differences in survival between groups were not calculated due to low number of patients in each group.

## Discussion

There are two major findings from our study. First, the 5-year-OS of patients with metastatic GCC has markedly improved compared to data from the 1997 IGCCCG classification system. The 5-year OS probabilities were 98% vs. 91%, 96% vs. 79%, and 66% vs. 48% for patients in the good-, intermediate- and poor-risk group, respectively. As we did not observe similar improvements in the PFS, the efficacy of first-line chemotherapy in metastatic GCC appears to be unchanged over the last decades. Indeed, while the substitution of etoposide for vinblastine as well as cumulative experience translated into improved survival rates in the early nineties (Williams et al. 1987; Gerl et al. 1996), a number of randomized controlled-trials on first-line chemotherapy published in the last two decades failed to demonstrate significant improvements in survival over standard BEP (Nichols et al. 1998; Motzer et al. 2007; Daugaard et al. 2011; de Wit et al. 2012; Fizazi et al. 2014; Necchi et al. 2015). By contrast, the improvement in the 5-years OS is likely due to salvage therapies that were given more consequently to a higher proportion of patients with relapsed GCC than this may have been the case some decades ago. In fact, standard first salvage regimens such as VIP, TIP or HDCT were applied as first salvage to the vast majority of relapsed patients if they did not undergo curative surgery for teratoma only (Kondagunta et al. 2005; Lorch et al. 2012; Adra et al. 2017). Further, adherence to structured follow-up schedules may have contributed to earlier diagnosis of GCC relapses (Albers et al. 2015; Cathomas et al. 2011).

Second, we observed no significant difference in the OS between good-risk and intermediate-risk patients. Thus, the IGCCCG prognostic score no longer predicts different survival probabilities for the groups of good and intermediate-risk patients. Similar data were recently reported from a Swiss cohort that included 204 patients treated from 1991 to 2016 (Fankhauser et al. 2018). As in our study, no significant difference in overall survival between good and intermediate risk patients was found. Another large retrospective observational study on 707 intermediate prognosis GCC patients reported a 5-year-OS rate of 89% in patients treated from 1997 to 2016, which was significantly superior to the 83% 5-years OS rate of 237 patients treated from 1979 to 1996 (Seidel et al. 2018). Of note, a lactate dehydrogenase (LDH) level > 2 upper limit of normal (UNL) and AFP levels of > 6200 IU/ml were independently associated with inferior survival in this study. Further, first-line treatment with three cycles of BEP was given to 53 (7%) patients with no significant differences found in OS and PFS between patients treated with three and four cycles of BEP. However, patient numbers were too low to draw any firm conclusion on whether de-intensification of treatment may be an option. In a subsequent analysis of the cohort intermediate risk patients with AFP values > 6000 IU/ml and LDH > 3 UNL revealed an outcome similar to the poor prognosis category (Seidel et al. 2019). Moreover, patients with AFP levels > 1982 IU/ml had a higher risk of disease recurrence (recurrence rate 20% vs. 31%; *P* = 0.02) as did patients with LDH levels > 2 UNL (recurrence rate 29% vs. 42.5%; *P* = 0.047). Thus, based on these findings and given the lack of data from prospective randomized trials, three cycles of BEP may only be used on an individual basis in intermediate risk patients with LDH level ≤ 2 UNL and AFP levels ≤ 1982 IU/ml (Seidel et al. 2018, 2019). A randomized controlled trial comparing three with four cycles of BEP in intermediate risk patients without high-risk features is warranted.

Our findings compare favorably with recent results from an analysis of a large data set collected to redefine the IGCCCG classification (Gillessen et al. 2019; Beyer et al. 2020). An international consortium contributed data on 9530 advanced non-seminoma GCC patients treated with cisplatin/etoposide based first line chemotherapy between 1990 and 2013. The 5-years OS was 96%, 89% and 67% for patients in the good-, intermediate- and poor-risk group, respectively (Gillessen et al. 2019). Further, based on data from 2302 advanced seminoma patients, 5-years OS rates were reported to be 95% and 87% for good- and intermediate-risk patients, respectively (Beyer et al. 2020). It should be noted that our data were also included in the IGCCCG project.

The 66% 5-years OS rate of poor-risk patients remains unsatisfactory although this represents an improvement compared to the original IGCCCG risk classification. However, attempts to improve overall survival by intensification of first line chemotherapy have failed (Motzer et al. 2007; Daugaard et al. 2011; Necchi et al. 2015), although a significant PFS-advantage was achieved by use of an intensified chemotherapy schedule for patients with inappropriate tumor marker decline (Fizazi et al. 2014).

Limitations of the present study are its retrospective design and the relatively small sample size. However, our analysis provides important real-life data on clinical outcomes of patients with metastatic GCC mostly managed outside a clinical trial setting.

In conclusion, compared to data from the original IGCCCG classification system, the outcome of patients with metastatic GCC has markedly improved. While the prognosis of intermediate-risk patients is excellent, treatment in the poor-prognosis group remains to be improved.

## Data Availability

The data that support the findings of this study are available on request from the corresponding author. The data are not publicly available due to privacy or ethical restrictions.
